# Exploring the relationship between loneliness, spirituality, and health-related quality of life in Hispanic cancer caregivers

**DOI:** 10.1007/s00520-022-06800-5

**Published:** 2022-02-10

**Authors:** Jennifer J. King, Chris Segrin, Terry A. Badger, Cynthia A. Thomson

**Affiliations:** 1 Department of Health Promotion Sciences, University of Arizona, Mel and Enid Zuckerman College of Public Health, 1295 N. Martin Ave., Drachman Hall, A260, PO Box: 245209, Tucson, AZ 85719 USA; 2 College of Social and Behavioral Sciences, University of Arizona, Tucson, AZ USA; 3 College of Nursing, University of Arizona, Tucson, AZ USA; 4 Department of Health Promotion Sciences and the University of Arizona Cancer Center, University of Arizona, Tucson, AZ USA

**Keywords:** Caregiver, Cancer, Hispanic, Loneliness, Spirituality

## Abstract

Caregivers of cancer patients find it challenging to perform their roles and to meet the demands of caregiving. Spirituality has been investigated as a potential coping strategy employed by caregivers, yet spirituality and related practices vary among cultural groups. In this study, we investigated the relationship between spirituality and health-related quality of life (HRQOL) and evaluated mediation effects of loneliness on this relationship. The sample was 234 lower socioeconomic status (SES) Hispanic caregivers of breast cancer survivors using existing data from the Support for Latinas with Breast Cancer and Their Intimate and Family Partners study, funded by the American Cancer Society (Badger, PI). A cross-sectional analysis was conducted at baseline, using self-reported spirituality, loneliness, and HRQOL data collected from 2012 to 2017. The exposures and outcomes were assessed using the Spiritual Well-Being Scale, the Social Isolation—Short Form 8a PROMIS Item Bank v2.0 scale, and the Global Health Scale PROMIS v.1.0/1.1 scale. Descriptive and mediation analyses using the Preacher and Hayes’ approach were conducted to estimate the direct effect of spirituality on HRQOL and the indirect effect of spirituality through mediation of loneliness in relation to HRQOL. A positive association between spirituality and HRQOL was found, whereas loneliness was inversely associated with HRQOL (*b* =  − .18, *SE* = .03, *p* < .0001). Age did not function as a moderator of the spirituality-HRQOL association in any of the models tested, but in the model testing mediation, loneliness was shown to mediate the association between spirituality and HRQOL (*b* =  − .17, *p* < .0001). These results suggest that spirituality may be beneficial to HRQOL in caregivers of Hispanic breast cancer survivors, due in part to reduced loneliness among more spiritual caregivers.

## Introduction

Caregiving has numerous adverse consequences related to the informal caregiver’s health, with caregivers reporting increased physical and psychological symptoms following cancer diagnosis [1]. Caregiving is burdensome; 40% of caregivers defined as family members or friends who provide unpaid supportive, informational, and emotional care [2] report high burden and 18% report medium burden [3]. Approximately 72% of caregivers of cancer patients spend an average of 33 h weekly with patients, and caregiving may involve complex medical care [3]. Caregivers frequently experience daily routine interruptions, excessive worry, and an overall increased burden while caring for cancer survivors. Evidence has shown that distress among cancer survivors is interdependent with the caregivers’ distress [4, 5]. Interestingly, caregivers report equal, if not higher, levels of emotional distress relative to the cancer survivors [6]. Similarly, the emotional distress of caregivers has negative implications for the cancer survivors’ quality of life (QOL). Caregivers regularly support cancer survivors emotionally while also providing complex medical and personal care in the home, but each caregiver’s unique level of distress can often inhibit their ability to provide the appropriate level of quality care [5]. Moreover, caregivers frequently lack adequate resources while learning to manage their own and the survivor’s symptoms [7].

To date, of the estimated 40 million family caregivers in the USA, 21% are Hispanic [8]. Each year, 149,100 new cancers are diagnosed in Hispanics, who are the fastest growing ethnic group in the USA [9]. Consequently, the demand for more caregivers is rising proportionately in the Hispanic population each year [10], frequently in the form of informal caring. [11]. Moreover, Hispanic and other minority caregivers tend to experience higher burdens from caregiving and spend more time on average providing care than their non-Hispanic giving counterparts [2]. Caregiving among Hispanics has been considered a culturally embedded value with Hispanic caregivers providing 45% of caregiving, which corresponds to 30 h per week in high burden caregiving time [2]. Consequently, Hispanic caregivers have an especially high risk of psychosocial, physical, and caregiver-associated burden [12, 13], and available studies report greater levels of distress and health disparities among this caregiving group [2]. These health disparities coupled with the demands of caregiving may create an unprecedented risk for low health-related quality of life (HRQOL) for Hispanic caregivers. Furthermore, informal caregivers regularly experiences increased responsibilities and complex demands that may compromise their HRQOL [14].

Loneliness is a psychosocial symptom of perceived social isolation (e.g., living alone, having no means of transportation, living in a rural area, or the subjective distressing feeling of being alone) that is associated with lower HRQOL and may mediate the relationship between spirituality and HRQOL in Hispanic caregivers of cancer survivors [15]. Importantly, caregiving can be associated with loneliness [12, 16] because the time demands of caregiving can reduce opportunities for socialization [17]. Loneliness and reduced social connections are known to promote stress among caregivers [18], including Hispanic adults [5], and can result in a lower HRQOL for both the Hispanic cancer survivors and their caregivers [19]. Loneliness appears to play a prominent role in burden experienced by caregivers of cancer patients [20–22]. This is likely due to the fact that in the context of cancer, caregiving can be increasingly consuming and in many cases is accompanied by anticipatory grief over the potential loss of the loved one. Spirituality is the relationship between God or a higher power and one’s self, while religiosity is the organizational component of attendance at meeting places related to a specific belief [23, 24]. Spirituality has been identified as an important dimension of quality of life (QOL). For Hispanics, health issues are often met with a spiritual approach of prayer and reliance on faith [25, 26]. Hispanics who regularly or even sporadically attend church are likely to identify prayer as important for the health of family members and their own personal health [27]. Regardless of religious attendance, Hispanics may attain health benefits from spiritual and religious beliefs, although some studies of Hispanic caregivers of family members with long-term disabilities report that intrinsic and organizational religiosity is associated with a lower perceived burden than non-organized religiosity [28]. Relevant Hispanic cultural values include Espíritu which is defined as trust in spirituality. Espíritu is an important Hispanic core cultural value in which individuals may find solace. Hispanics report frequent engagement in spiritual activities as a way to cope with life events that they associate with stress [29, 30].

The current study was designed to explore the relational aspect of spirituality that may reduce perceptions of loneliness [31] while examining how that benefit is indirectly associated with overall HRQOL among Hispanic breast cancer caregivers. The research aims were to determine the total influence, if any, of spirituality on the HRQOL and loneliness in Hispanic breast cancer caregivers and to determine the extent to which perceived loneliness mediates the effect of spirituality on HRQOL. Our hypothesis of this investigation was that Hispanic cancer caregivers reporting higher spirituality would report lower perceived loneliness and higher HRQOL. Additionally, loneliness is hypothesized to mediate the relationship between spirituality and HRQOL. Hawkley and Cacioppo’s theoretical model of health and loneliness offers a useful framework for this investigation and explicates how loneliness accelerates the age-related deterioration of health [32–34]. The process of aging is naturally associated with declining health in many people. Hawkley and Cacioppo’s model specifies mechanisms that are adversely affected by loneliness (e.g., stress responses, corruption of recuperative behaviors, diminished health behaviors) that exaggerate the effect of age on health-related quality of life; because cancer, loneliness and spirituality each increase with age [35, 36], we additionally sought to evaluate age as a moderator of the relationships among these factors.

## Methods

### Parent study

The parent study utilized a randomized clinical trial design to compare improvements in QOL potentially resulting from two 8-week interventions: (1) a telephone-delivered counseling intervention and (2) a supportive health education intervention. Inclusion criteria for the caregivers in the parent study [37] were (1) nominated by a Hispanic woman with breast cancer; (2) over 21 years of age; (3) English- or Spanish-speaking; and (4) had access and ability to talk on the telephone. The caregivers were defined as any individual within the naturally occurring support system whom the survivor chose to participate in the study, whether or not the person was related by blood or marriage or cohabitating. Two types of caregivers were as follows: (1) intimate partners were defined as spouses/significant others in an intimate relationship with the survivor; (2) other family members/friends were defined as any person related by blood or emotional attachment (other family relative or friend but was not a spouse or intimate partner). Of the total caregivers, 91% (234) were of Hispanic descent. For the purposes of this secondary analysis, only caregivers who reported their ethnicity as Hispanic were included.

### Measures

At baseline, all participants were asked to complete a self-report of spirituality and loneliness which was assessed by the Spiritual Well-Being Scale and the Social Isolation—Short Form 8a PROMIS Item Bank v2.0 scale. HRQOL was assessed using the Global Health Scale PROMIS v.1.0/1.1 scale.

*Loneliness* was measured by the Social Isolation—Short Form 8a PROMIS Item Bank v2.0: “I feel left out”; “I feel that people barely know me”; “I feel isolated from others”; “I feel that people are around me but not with me”; “I feel isolated even when I am not alone”; “I feel that people avoid talking to me”; “I feel detached from other people”; and “I feel like a stranger to those around me.” All the questions follow the Likert scale from 1 (*never*) to 5 (*always*).

*Spirituality* was measured by the Spiritual Well-Being Scale that contains items such as “How important to you is your participation in religious activities such as praying, going to church or temple?”; “How important to you are other spiritual activities such as meditation and praying?”; “Do you sense a purpose/mission for your life or a reason for being alive?”; “How much uncertainly do you feel about the future?”; and “How hopeful do you feel?” Although we refer to this measure as “spirituality” following the title of the scale, in actuality, it assesses an amalgamation of spirituality and religiosity. All the questions follow the Likert scale from 1 (*not at all important*) to 10 (*very important*).

*HRQOL* was measured with four items from the 10-item PROMIS Global Health Scale v.1.0/1.1: GH1: “In general, would you say your health is”; “In general, would you say your quality of life is”; “In general, how would you rate your physical health”; and “In general, how would you rate your mental health, including your mood and your ability to think”. These questions have a Likert scale that starts at 1 (*poor*), 2 (*fair*), 3 (*good*), 4 (*very good*), up to 5 (*excellent*). Items are summed for a total possible score range of 7–70 for spirituality, 8–40 for loneliness, and 4–20 for HRQOL.

### Statistics analysis

**Fig. 1:**
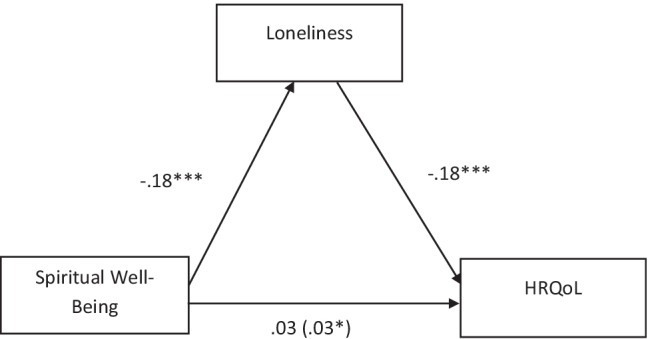
Mediation: general health, spiritual well-being, and loneliness. Note. Figure values are unstandardized regression coefficients. Coefficient in () is the indirect effect. * *p* < .05. *** *p* < .001

**Fig. 2:**
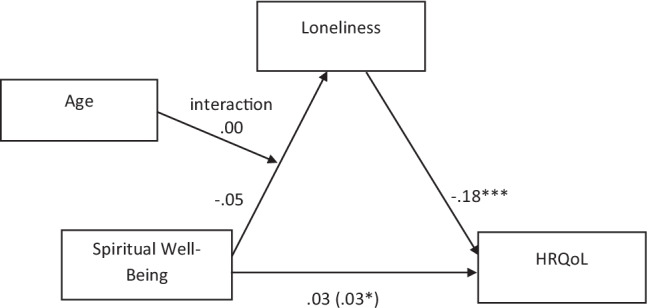
Moderated mediation: indirect effect of spirituality on perceived health varies as a function of age, where age is moderating the path from spirituality to loneliness. Note. Figure values are unstandardized regression coefficients. Coefficient in () is the indirect effect. * *p* < .05. *** *p* < .001

**Fig. 3:**
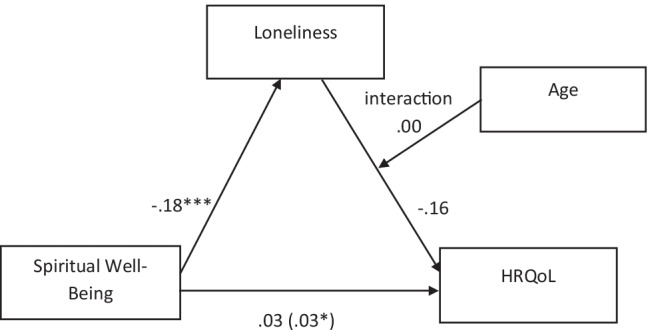
Moderated mediation: indirect effect of spirituality on health varies as a function of age, where age is moderating the path from loneliness to perceived health. Note. Figure values are unstandardized regression coefficients. Coefficient in () is the indirect effect. * *p* < .05. *** *p* < .001

Descriptive statistics were calculated to examine key characteristics of 234 caregivers of Hispanic women with breast cancer. Three different regression-based models were employed that varied only by the specification of the path moderated by caregiver’s age, if any. In all three models, HRQOL was the dependent variable (DV), self-reported spirituality was the independent variable (IV), and loneliness was the mediating variable (M). First, we tested a mediation model without any moderated paths (Fig. 1). In the first step, loneliness (M) was regressed on spirituality (IV). Second, we tested whether the indirect effect of spirituality on perceived health varies as a function of age, where age is moderating the path from spirituality to loneliness (Fig. 2). Finally, we tested whether the indirect effect of spirituality on health varies as a function of age, where age is moderating the path from loneliness to perceived health (Fig. 3). The effective sample size for the mediation analyses was 232. Mediation was tested using the Preacher and Hayes’ approach to estimate direct and indirect effects of spirituality on HRQOL through the mediator of loneliness. A bias-corrected bootstrapping analytic procedure based on 5000 bootstrap samples to estimate confidence intervals (CIs) around the indirect effect of spirituality on HRQOL was applied. Three models were tested with a 95% CI around the indirect effect. The indirect relationship between spirituality on HRQOL through loneliness in Hispanic cancer caregivers was tested for moderation as a function of survivors’ age.

## Results

Table 1 summarizes the demographic characteristics of the breast cancer caregiver participants. Caregivers were mostly females of lower socioeconomic states (SES) who were married, self-identified as Mexican American, with high school, vocational, technical, or some college education, and who were employed full-time. The mean age of caregivers in the study was 50.6 years; 7.6% were ≥ 65 years of age. The majority was female relatives of the breast cancer survivor and in self-described “moderate health” HRQOL (mean: 12.68 ± 3.13), “lower” loneliness (mean: 13.02 ± 6.31), and of “higher” spirituality (mean: 54.65 ± 9.42).

**Table 1:** Baseline characteristics of sample

Variable	*(N* = *234) Mean (SD) or N (%)*
Age	50.6 (10.3)
Sex of participant (female)	150 (64%)
Ethnicity	
Mexican/Mexican American	119 (51%)
Hispanic/Latina	73 (31%)
South American	8 (3%)
Central American	5 (2%)
Marital status	
Married	153 (65%)
Unmarried	68 (29%)
Caregiver is	
Spouse, significant other	66 (28%)
Child	46 (19%)
Mother	36 (15%)
Cousin	2 (.9%)
Sibling	35 (15%)
Friend	24 (10%)
Other	14 (6%)
Education	
Elementary	21 (9%)
Middle school	42 (18%)
High school	59 (25%)
Vocational/technical school or some college	61 (26%)
College	33 (14%)
Post graduate or professional	6 (3%)
Other	1 (< 1%)
Employment	
Full-time	96 (41%)
Unemployed, seeking	33 (14%)
Part-time	29 (12%)
Retired	21 (9%)
Disabled	8 (3%)
Other	36 (15%)

^*^N available

Table 2 presents a correlation matrix of all the study variables. An evaluation of the relationship between spirituality and separately loneliness and HRQOL showed that those with higher spirituality, regardless of age, reported higher HRQOL (Fig. 1). There was a significant mediating effect of loneliness on HRQOL with the lonelier participants reporting worse health (*b* =  − 0.17, *p* < 0.0001). Greater reported loneliness was also associated with lower, self-reported HRQOL.

**Table 2:** Correlation matrix of study variables: general health, spiritual well-being, loneliness, and age

	1	2	3	4
1. General health	–			
2. Spiritual well-being	.16*	–		
3. Loneliness	− .36**	− .19**	–	
4. Age	− .07	.02	.04	–

*Note.* *Correlation is significant at the 0.05 level (2-tailed) *p* < .05. **Correlation is significant at the 0.01 level (2-tailed) *p* < .01

### Mediation analyses

To further test these relationships, mediation and moderated mediation analyses were performed. To investigate whether the relationship between self-reported spirituality and HRQOL in caregivers of Hispanic cancer survivors could be explained by self-reported loneliness, we also explored several moderated mediation models in relation to age of caregivers. As hypothesized, spirituality was significantly and negatively associated with loneliness (*b* =  − 0.18, SE = 0.05, *p* = 0.0002), such that higher endorsement of spirituality was associated with reduced loneliness. Testing mediation without any moderated paths (Fig. 1), loneliness was regressed on spirituality (IV). In this analysis evaluating perceived health (DV) in relation to spirituality (IV) and loneliness (M), loneliness was shown to be a significant predictor of health (*b* =  − 0.18, *SE* = 0.03, *p* < 0.0001). The direct effect from spirituality to health was not significant (*b* = 0.03, *SE* = 0.02, *p* = 0.18). However, the indirect effect of spirituality on health through loneliness was significant (*b* = 0.03, bootstrap *SE* = 0.01, bootstrap 95% CI: 0.01–0.06).

### Moderated mediation analyses

Results for the analysis of moderation by caregiver’s age (Fig. 2) showed that when loneliness was regressed on spirituality, age, and the interaction, neither of the main effects nor the interaction term was significant, suggesting that age does not moderate the relationship between spirituality and loneliness. The index of moderated mediation was not significantly different from 0 (95% bootstrap CI: − 0.0009 to.0019), indicating that the indirect effect of spirituality on health is not conditional on age.

Interestingly, when we tested whether the indirect effect of spirituality on health varies as a function of age, where age is moderating the path from loneliness to perceived health (Fig. 3), age did not moderate the relationship. In the first step, loneliness was regressed on spirituality only (IV-M path as in model 4), confirming that lower endorsement of spirituality was associated with higher loneliness. In the second step, perceived health was regressed on spirituality, loneliness, age, and the interaction between loneliness and age. None of the effects in this regression model were significant. These results suggest that age does not moderate the relationship between loneliness and perceived HRQOL.

In summary, across all three models, the direct effects of spirituality on health were not significant; the indirect effects of spirituality on health via loneliness were significant. There were no moderating effects of age for any of the two components of the indirect effect that we tested (spirituality to loneliness and loneliness to perceived health).

## Discussion

The purpose of this study was to explore the influence of spirituality on the HRQOL and loneliness in caregivers who were shown to be of lower SES Hispanic breast cancer survivors. Major findings indicate that there was a significant association between spirituality and HRQOL such that the people with better spirituality reported better health. In the model test of mediation, with loneliness as the mediator, a significant direct effect was shown between loneliness and HRQOL. An indirect effect of spirituality on higher HRQOL via reduced loneliness was demonstrated. Our main finding suggests spirituality may be beneficial to HRQOL, due in part to reduced loneliness among more spiritual caregivers. These effects appear to be consistent over the lifespan because age did not moderate the effects of spirituality in Hispanic cancer caregivers. Prior work has established the relationship between patient HRQOL and loneliness [38], but the influence of the patient caregiver’s spiritual well-being upon this relationship, as demonstrated here, has not previously been examined. With loneliness and HRQOL shown to be interrelated [32, 39], these findings indicated similar significant results, further informing on the nature of loneliness influences on HRQOL and potential pathways to alleviate poorer health outcomes via spirituality. In this mediational relationship, as self-reported loneliness decreased and spirituality increased, HRQOL improved.

These findings also showed that caregiver loneliness played a significant role as a mediator in the relationship between caregiver Spiritual Well Being (SWB) and HRQOL, which is the first demonstration of this effect among Hispanic cancer caregivers reported in the literature. Two life aspects of interest, loneliness and spirituality, and the association with HRQOL were significant. Research has been shown to elucidate these models in prior studies among cancer survivors, but limited work has been done among caregivers, especially in Hispanics [40, 41]. While survivors were not the focus of this study, our finding suggested that the nature of the relationships between SWB, loneliness, and HRQOL among Hispanic cancer caregivers were similar to other studies where these are significant relationships to consider for dyadic outcomes [39, 42]. Our study results were comparable to findings that demonstrated caregiver spirituality is central to coping strategies and it is a buffer that influenced caregiver HRQOL [43–45]. Of note in the present study, this relationship remained stable and statistically significant over a range of ages, in contrast to other findings [46, 47]. However, it should be noted that less than 15% of the present sample was over the age of 60 years, which is a point in the lifespan where age-related changes in health would begin to become most evident. It is at least possible that in a sample of caregivers in older age, the hypothesized moderating effect of age on the loneliness-spirituality-health mediation could be more apparent.

The findings from this study showed that the generally positive association between spirituality and better HRQOL may be explained in part by the lower levels of loneliness—a phenomenon that is deleterious to health—among those with higher levels of spiritual well-being. Accordingly, the most effective intervention to promote higher HRQOL for Hispanic caregivers may well be to attend to the caregiver spiritual needs if they have demonstrated experiences of loneliness [40]. Interventions that include spiritual counseling, programs, or therapy to enhance spiritual well-being among caregivers of Hispanic cancer survivors should be explored [48]. Additional research is needed to more robustly evaluate the impact of spirituality in Hispanic cancer caregivers and what role loneliness has in potentially influencing HRQOL.

Certainly, spirituality is as an important dimension of QOL and may be particularly so for Hispanics who often practice spiritual approaches to address health issues in addition to relying on faith. Our findings provide a rationale for future studies that include religious and spiritual approaches to support Hispanic cancer caregivers’ QOL[25, 26]. As reported, Hispanics who regularly attend church and those who attend only sporadically are equally likely to identify prayer as important for family and personal health [27]. Therefore, regardless of the frequency of religious attendance, Hispanics may attain health benefits from spiritual and religious beliefs. Given all the challenges, caregivers remain a fundamental source of care for cancer patients in the USA, yet the psychosocial needs and health outcomes of caregivers are less well understood, especially among Hispanic caregivers. Caregivers of cancer patients play an important role in the patient’s every day care management and end-of-life care [49, 50]. Caregivers with increased stress due to the extended length of caregiving have demonstrated an increased risk of morbidity and mortality [51]. Greater time, effort, and resources placed into caregiving are associated with compromised social life, further deteriorating a caregiver’s QOL and adversely affecting health [52]. The growing population of survivors and caregivers warrants further investigation into the roles and demands of caregiving, especially among minority populations.

## Limitations

There are limitations to the present study that should be considered when interpreting its findings. First, the study included only caregivers of Hispanic women with breast cancer; therefore, it is unclear whether the results would be generalizable to the larger population of non-Hispanic caregivers. Given the lower socioeconomic status of this sample and the literature documenting that spirituality increases for those with lower SES and minorities, these results cannot be generalized to those with higher SES and non-Hispanic Whites [53]. Second, the data used for testing the hypotheses were cross-sectional, and this cannot substantiate causal connections between the independent, mediating, and dependent variables as convincingly as longitudinal data. Finally, most of the sample were middle aged (< 60 years of age), limiting the ability to robustly evaluate age-related influences of QOL, spirituality, and loneliness. Future research that examines the nature of the relationships between caregiver HRQOL, loneliness, and spirituality with different caregiver populations, including those of a broader age range, will add to the generalizability of study results.

## Conclusion

Our principal finding is that higher spirituality has an indirect effect on better health-related quality of life through reduced loneliness among Hispanic cancer caregivers. With the burden of higher mortality after cancer in Hispanics and concerns over poor health-related quality of life, our findings suggest that it will be important to address spirituality and loneliness among informal caregivers. This is consistent with Hawkley and Cacioppo’s (2007) model of health and loneliness which posits that lower perceptions of loneliness predict better health outcomes. It would appear that this study further clarifies this relationship by showing an association between spirituality and loneliness. This illustrates an area for further investigation, and possibly interventions addressing spirituality as well as loneliness, to improve caregiver outcomes. Specifically, interventions that provide strategies for improving spirituality, such as teaching spiritual self-care, facilitating a regularly designated time for spiritual activities (e.g., explore engagement in spirituality-based services, practices, readings or identify alternative approaches to enhance HRQOL among caregivers with lower spirituality), and finding purpose and meaning in life, can improve HRQOL. Given these approaches pose no side effects or risks, are low cost, and are readily available, acquiring necessary supportive spiritual skills may help to alleviate poorer caregiver HRQOL related to caregiving demands for family members who are cancer survivors. This will ultimately help maintain the caregiver’s own health and quality of life enhancing the ability and opportunity for a caregiver to deliver effective care to survivors.

## Data Availability

N/A.

## Code availability

N/A.
